# Hematological and neurological expressed 1-mediated anoikis resistance promotes anaplastic thyroid cancer metastasis

**DOI:** 10.1016/j.gendis.2025.101558

**Published:** 2025-02-19

**Authors:** Huangcan Li, Simiao Fan, Zhongqin Gong, Chunlei Nie, Jiangqi Liu, Lingbin Xue, Xianhai Zeng, Jason Ying Kuen Chan, Michael Chi Fai Tong, George Gong Chen

**Affiliations:** a Guangzhou Municipal and Guangdong Provincial Key Laboratory of Molecular Target & Clinical Pharmacology, The NMPA and State Key Laboratory of Respiratory Disease, School of Pharmaceutical Sciences, Guangzhou Medical University, Guangzhou, Guangdong 511436, China; b Department of Otorhinolaryngology, Head and Neck Surgery, Faculty of Medicine, Chinese University of Hong Kong, Hong Kong, China; c Department of Head and Neck Surgery, Harbin Medical University Cancer Hospital, Harbin, Heilongjiang 150081, China; d Department of Otolaryngology, Longgang E.N.T Hospital and Shenzhen Key Laboratory of E.N.T, Institute of E.N.T Shenzhen, Shenzhen, Guangdong 518116, China; e CUHK-Shenzhen Otolaryngology and Head and Neck Institute, Shenzhen Research Institute (SZRI), Shenzhen, Guangdong 518057, China

The plasticity of cancer cells enables them to adapt to selective pressures, further enhancing their survival advantage and resistance to apoptosis. Cancer cell plasticity operates through multiple mechanisms. Among them, the key to successful cancer metastasis mainly depends on epithelial–mesenchymal transition (EMT) and anoikis resistance. EMT enables cancer cells to acquire migratory and invasive capabilities, allowing them to overcome tissue barriers and adapt to new environments.^1^ Anoikis resistance enables detached cancer cells to circumvent the apoptotic response that occurs upon detachment from their normal extracellular matrix environment.^2^ Hematological and neurological expressed 1 (HN1) has been shown to influence integrin-mediated cell adhesion and signaling, critical for cell-extracellular matrix interactions and anchorage-dependent cell survival.^3^ HN1 can determine the survival of cancer cells under conditions of extracellular matrix adhesion changes through integrins. These effects further validate the importance of HN1 in the regulation of cancer cell survival and metastasis. Though HN1 acts as an EMT regulator or mediator for downstream signaling pathways in different cancers, the role of HN1 in anoikis resistance has not been reported before.

To verify the significance of anoikis resistance in anaplastic thyroid carcinoma (ATC), we initially conducted a comparative analysis of cell viability among normal thyroid cells, papillary thyroid carcinoma cells (B-CBAP), and ATC cells (KAT 18) using the established anoikis-inducing agent tetrathiomolybdate (TM). The findings revealed that KAT 18 cells exhibited a high tolerance to over 100 times the concentration of TM (IC_50_ = 352.38 ± 15.41 μM) compared with normal thyroid cells (IC_50_ = 28.04 ± 0.59 μM), or 3 times more than B-CBAP cells (115.65 ± 11.28 μM), underscoring a robust resistance to anoikis in ATC. Next, we further verified the expression level of HN1 from thyroid tissues collected from different types of thyroid cancer patients and different thyroid cancer cells (Fig. 1A–C). We confirmed that HN1 was highly expressed in ATC compared with papillary thyroid cancer or normal thyroid cells. Both translational and transcriptional analyses confirmed an elevation in HN1 expression following TM treatment, indicating its critical role in the context of cell detachment (Fig. 1D, E; Fig. S1A, B). Live cell imaging of KAT 18 cells expressing GFP-HN1 after TM treatment revealed a gradual increase in HN1 signal intensity, with over 60% of surviving cells showing notable HN1 expression after 24 h (Fig. 1F).

**Figure 1:**
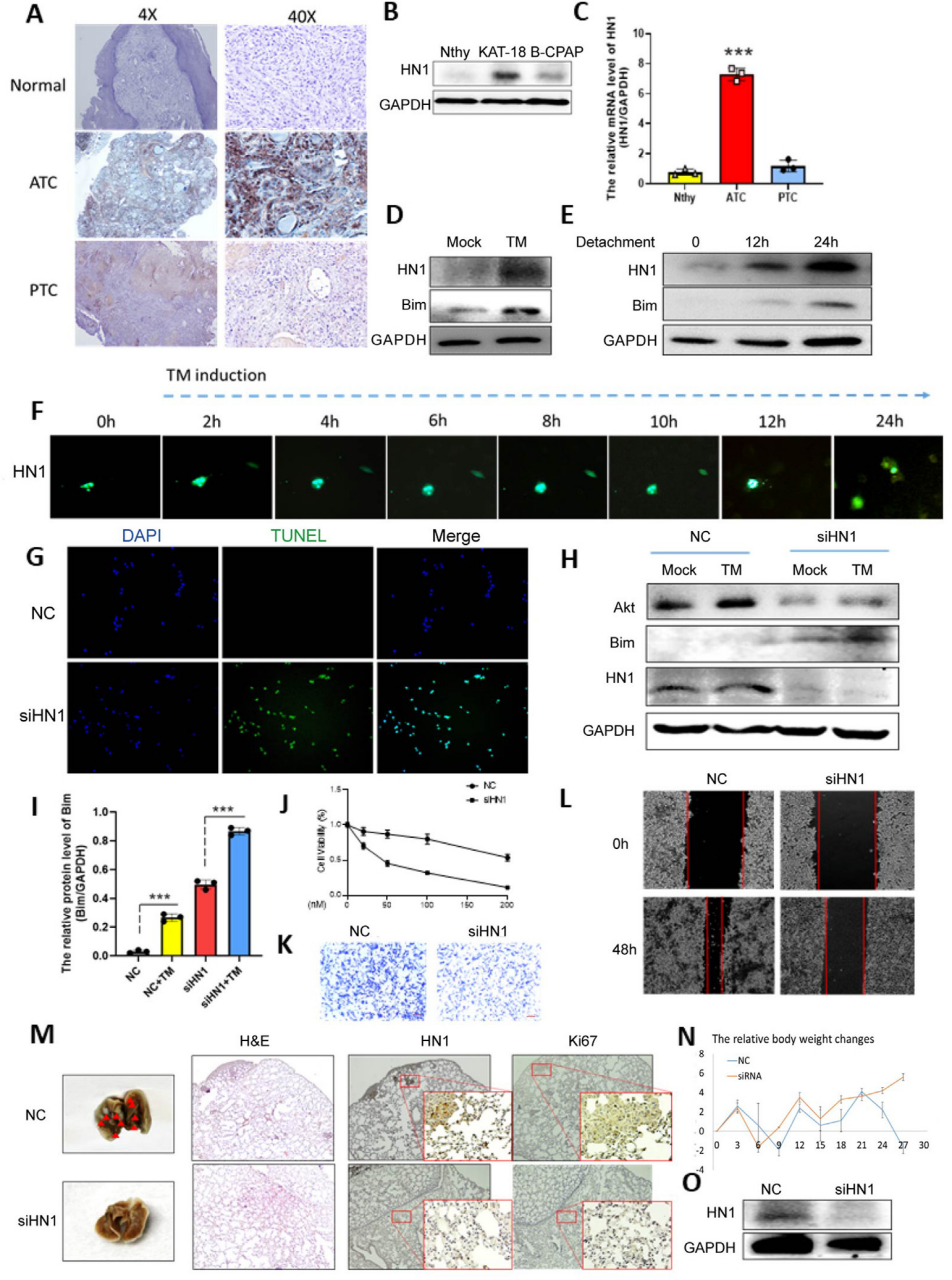
Hematological and neurological expressed 1 (HN1) promotes anoikis resistance in anaplastic thyroid cancer (ATC). **(A)** Immunohistochemistry staining of HN1 from normal thyroid tissues, ATC tissues, and papillary thyroid cancer (PTC) tissues. **(B, C)** Immunoblots (B) and quantitative PCR analysis (C) of lysates among normal thyroid cells (Nthy), PTC line B-CBAP, and ATC cell line KAT-18 with the HN1 expression. **(D, E)** Immunoblots of lysates from KAT-18 cells with tetrathiomolybdate (TM) treatment (D) or detachment culture (E) for 24 h. **(F)** KAT-18 cells were transfected with GFP-HN1. The live images from GFP-HN1 expressing KAT-18 cells after 0–24 h TM treatment. The field of view is fixed to the area for tracking and recording. **(G)** Anoikis assay was detected by TUNEL staining. KAT-18 cells were transfected with siRNA negative control (NC) or siRNA-HN1 (siHN1) for 48 h, and then the cells were treated with or without TM for 24 h. **(H, I)** Immunoblots of lysates from KAT-18 cells (NC) or HN1-knockdown KAT-18 cells (siHN1) induced with mock buffer or TM for 24 h (H). Densitometric analysis of Bim (I) levels was conducted and the levels were normalized with GAPDH using ImageJ. **(J)** MTT assay for comparing the effect of sorafenib in KAT-18 transfected with or without siRNA-HN1. **(K, L)** Trans-well (K) and wound healing assays (L) were employed to analyze the correlation of HN1 with the invasion and migration of ATC cells. **(M)** The bright field image, hematoxylin-eosin staining, and immunohistochemistry staining analysis of the lung harvested from mice after different treatments. The red arrows point out the nodules on the lungs. **(N)** The relative body weight changes within 28 days. **(O)** Immunoblots of lysates from the lung tissues. Results were expressed as mean ± standard deviation (error bars) of three repeats. ∗*P* ≤ 0.05, ∗∗*P* ≤ 0.01, ∗∗∗*P* ≤ 0.001 (unpaired *t*-test).

Given the strong correlation between HN1 and anti-apoptotic processes, we postulated that HN1 significantly influences anoikis resistance in ATC. To verify this assumption, we cultured KAT 18 cells on detachment plates and analyzed apoptosis using the TUNEL assay (Fig. S1C, D). Normal thyroid cells exhibited low survival rates during suspension culture, while KAT-18 cells demonstrated strong anoikis resistance. Notably, depletion of HN1 increased anoikis sensitivity by over six-fold, confirming the essential role of HN1 in promoting anoikis resistance. Immunofluorescence imaging further illustrated that silencing HN1 led to increased apoptosis in these anoikis-resistant cells (Fig. 1G; Fig. S1E). This suggests that HN1 is vital for supporting cell survival under detachment conditions.

A previous study has identified key signaling pathways, including epidermal growth factor receptors (EGFR) and phosphatidylinositol 3' -kinase (PI3K)/protein kinase B (Akt), as crucial players in anoikis resistance across various cancer types. Meanwhile, the ectopic expression of HN1 triggers Akt to activate lipogenesis, thereby promoting metastasis in hepatocellular carcinoma.^4^ Therefore, we hypothesized that ATC cells may also resist anoikis response by the elevation of the HN1-Akt axis. To verify this assumption, we then examine the expression changes of HN1 and Akt in ATC cells under anoikis assay. Transfecting cells with siRNA targeting HN1 resulted in decreased Akt levels and increased expression of the anoikis marker Bim, particularly in the presence of TM. This indicates a negative relationship between HN1 and Bim while suggesting a positive correlation between HN1 and Akt (Fig. 1H, I; Fig. S1E, F). Since anoikis resistance contributes to cancer metastasis by empowering cancer survival, we next investigated whether HN1 could assist cancer migration and invasion after detachment. Since sorafenib is broadly used for limiting thyroid cancer metastasis, we first confirmed that the depletion of HN1 increased the effect of sorafenib on ATC (Fig. 1J), indicating its high potential in cancer treatment. To further examine the treatment effect on cancer metastasis, ATC cells were transfected with siRNA targeting HN1 for trans-well and wound-healing assays (Fig. 1K, L; Fig. S1H–M). Consistent with the previous results, HN1 knockdown inhibits invasion and migration in ATC. Collectively, these results implied a promising approach in the treatment of cancer metastasis by targeting HN1-mediated anoikis resistance.

To explore HN1's effects on metastatic cells *in vivo*, we injected HN1-depleted KAT 18 cells into the tail vein of mice. After two weeks, the control group exhibited significant weight loss and severe lesions in the lungs and liver, as confirmed by hematoxylin-eosin staining analysis (Fig. 1M, N). The number of lung nodules was higher in the control group compared with the siHN1 treatment group. As shown in the immunohistochemistry staining, the lung without apparent nodules expressed less HN1. To figure out whether the expression of HN1 affects tumor formation, the identical lung tissue from mice bearing with or without nodules was collected and subjected to Western blot. The result indicated that the higher expression level of HN1 is correlated with the cases of nodule formation (Fig. 1O). Among the various sites of metastasis in ATC, the lung represents the most common destination for disseminated ATC, contributing significantly to disease progression and poor patient prognosis. Therefore, it is reasonable to conclude that HN1 knockdown can suppress ATC metastasis.

Previously, HN1 was revealed to play multifaceted roles in promoting cancer cell survival and metastasis, highlighting its potential as a key therapeutic target in oncology. Although the regulation of HN1 in the EMT pathway has been mainly discussed, our current findings extend its significance to the realm of anoikis resistance, particularly in ATC. Compared with the lower expression in normal cells and other mild thyroid cancers, the high expression of HN1 makes subsequent intervention against ATC, a malignant thyroid cancer, simpler and more accurate. Both *in vivo* and *in vitro* experiments have preliminarily confirmed that controlling the expression of HN1 can effectively reduce the plasticity and increase its environmental sensitivity in ATC, thereby achieving the effect of inhibiting cancer metastasis. Mechanistically, we found that HN1 suppressed anoikis through Akt. Previous studies have confirmed that anoikis is controlled by the expression of EGFR on the cell membrane.^5^ Since HN1 is a downstream effector of EGFR, we hypothesized that the increase of EGFR in ATC cells under the influence of the detachment environment leads to HN1 inhibiting the downstream anoikis reaction. Meanwhile, we found that reducing HN1 in ATC cells increased the sensitivity of cancer cells to anti-cancer drug treatment, thereby promoting ATC cell death and impeding their metastatic activity. By synthesizing our current data with insights from existing research, it is imperative to elucidate the interactions among key cancer signaling pathways mediated by HN1, as this holds significant research implications and clinical relevance.

In summary, this initial data highlights that HN1 promotes anoikis resistance by regulating the Akt and Bim expression. This study further deepens the understanding of the key role of HN1 in cancer metastasis and will help develop new therapeutic strategies aimed at patients with ATC malignancies.

## CRediT authorship contribution statement

**Huangcan Li:** Manuscript Original Draft, Reviewing and Editing, Obtaining Experimental Results, Data Analysis, and Conceptualization. **Simiao Fan:** Manuscript Original Draft, Obtaining Experimental Results, and Validation. **Zhongqin Gong:** Data Analysis and Curation. **Chunlei Nie:** Data Curation and Clinical Data and Sample Collection. **Jiangqi Liu:** Clinical Sample Collection and Storage. **Lingbin Xue:** Methodology. **Xianhai Zeng:** Resources. J**ason Ying Kuen Chan:** Resources. **Michael Chi Fai Tong:** Supervision, Conceptualization and Funding Acquisition. **George Gong Chen:** Manuscript Reviewing and Editing, Supervision, Conceptualization and Funding Acquisition

## Ethics declaration

All animal experiment protocols were approved by the Ethics Committee of the Chinese University of Hong Kong with the license from the Department of Health, Hong Kong Special Administrative Region, China (Ref Number: (23-1317) in DH/HT&A/8/2/1 Pt.57).

## Funding

This study was supported by grants from the Research Grants Council of the Hong Kong Special Administrative Region, China (No. CUHK 14108921) and the 10.13039/501100001809National Natural Science Foundation of China (No. 82402076).

## Conflict of interests

The authors declared no competing interests.
