# Supplementary material for: Hematological and neurological expressed 1-mediated anoikis resistance promotes anaplastic thyroid cancer metastasis
Source: Genes Dis. 2025 Feb 19;13(2):101558. doi: 10.1016/j.gendis.2025.101558 (PMC12594924; doi:10.1016/j.gendis.2025.101558)
Supplement: Multimedia component 1 [file mmc1.docx]

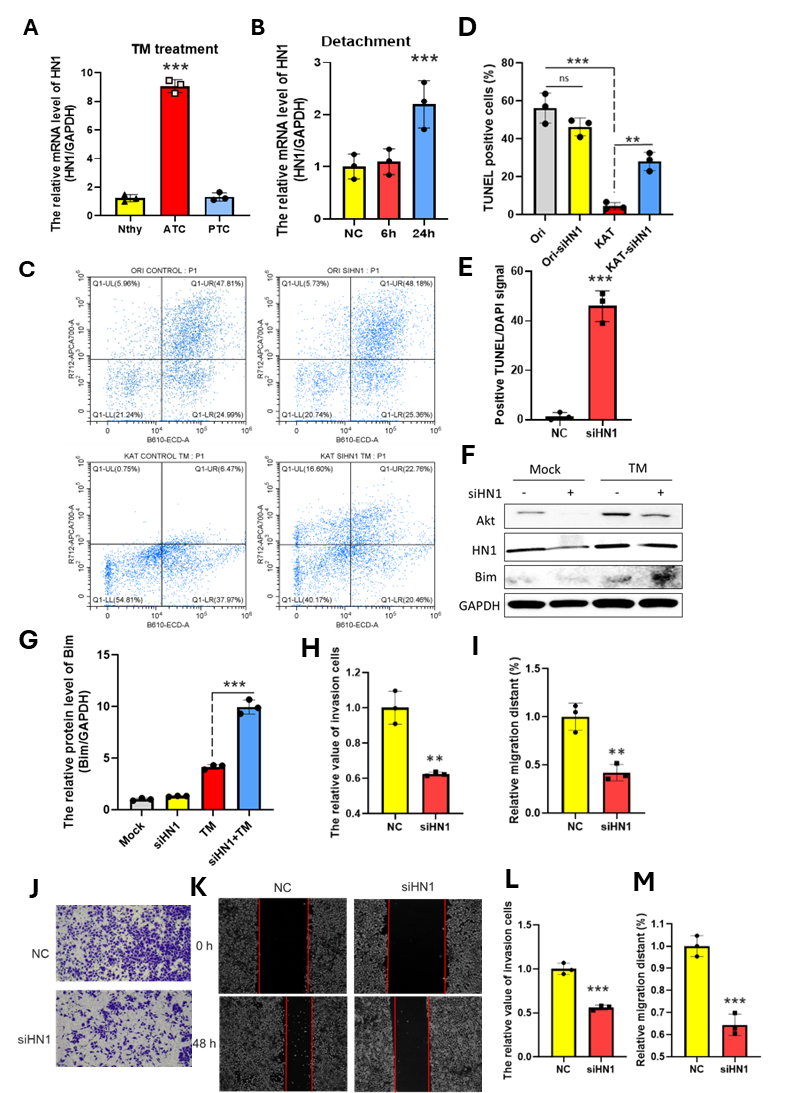


Supplementary Figure 1. **A.** The mRNA level of HN1 from different thyroid cells after 24h TM treatment. **B.** mRNA levels from KAT-18 cells after detachment within 24 h. **C-D.** Anoikis assay analysis by flow cytometry (C). TUNEL positive cells were then compared among normal thyroid cells and high anoikis resistance thyroid cancer cells, KAT-18 (D). E. The positive signal of TUNEL assay after 24h TM treatment in KAT-18 cells. **F-G.** The indicated protein expression level in 8505C after different treatment. 8505C cells were transfected with siNC (NC) or siRNA-HN1 for 48 h, then cells were pre-treated with mock buffer or TM for another 24h. Lysates were harvested and subjected to western blotting analysis (F). Densitometric analysis of Bim (G) levels was quantified and normalized with GAPDH using ImageJ. **H.** The relative invasion values ​​of HN1 knockdown KAT-18 cells or control cells after TM treatment. **I.** The wound healing effect in KAT-18 cells was determined by the relative migration distant. **J-M.** Trans-well (J) and wound healing assays (K) were employed to analyze the correlation of HN1 with the invasion and migration of ATC cells 8505C. The relative invasion values ​​of HN1 knockdown 8505C cells or control cells after TM treatment (L). The wound healing effect was determined by the relative migration distant (M). Results were expressed as mean ± standard deviation (error bars) of three repeats. *P ≤ 0.05, **P ≤ 0.01, ***P ≤ 0.001 (unpaired t-test).

|  | sense（5'-3'） | antisense（5'-3'） |
| --- | --- | --- |
| HN1 | TCAAGCTTCTTGGGCCAA | TTGGCCCAAGAAGCTTGA |
| Bim | CAAGAGTTGCGGCGTATTGGAG | ACACCAGGCGGACAATGTAACG |
| E-cadherin | GCCTCCTGAAAAGAGAGTGGAAG | TGGCAGTGTCTCTCCAAATCCG |

Supplementary Table 1 The primers of the indicated genes.
